# Known and New Emerging Viruses Infecting Blueberry

**DOI:** 10.3390/plants10102172

**Published:** 2021-10-14

**Authors:** Norsazilawati Saad, James W. Olmstead, Jeffrey B. Jones, Arvind Varsani, Philip F. Harmon

**Affiliations:** 1 Department of Plant Pathology, University of Florida, Gainesville, FL 32611, USA; jbjones@ufl.edu; 2 Department of Plant Protection, Faculty of Agriculture, Universiti Putra Malaysia, Serdang 43400, Malaysia; 3 Horticultural Sciences Department, University of Florida, Gainesville, FL 32611, USA; james.olmstead@driscolls.com; 4 The Biodesign Center of Fundamental and Applied Microbiomics, School of Life Sciences, Center for Evolution and Medicine, Arizona State University, 1001 S. McAllister Ave, Tempe, AZ 85287, USA; arvind.varsani@asu.edu; 5 Structural Biology Research Unit, Department of Integrative Biomedical Sciences, University of Cape Town, Cape Town 7925, South Africa

**Keywords:** *Vaccinium*, blueberry, viruses

## Abstract

Blueberry (*Vaccinium* spp.) plants are exposed to existing and emerging viruses as a result of expanding acreage of blueberry plantations across the world, primarily in North America. Since blueberry is cultivated in areas where there are wild *Vaccinium* spp., there is increasing risk of virus movement between wild and cultivated blueberries. This is theoretically possible because viruses can spread from commercial cultivars to native species and vice versa causing the spread of existing and new viruses. The occurrence of these viruses in blueberry can be devastating to the industry considering the cost for cultivation and production of this perennial crop. However, the advent of high-throughput sequencing and bioinformatic sequence analysis have allowed for rapid identification of known and novel viruses in any crop including blueberry, thus facilitating proper intervention in response to serious viral diseases. In this paper, we aim to focus on the current status of known and novel viruses emerging in blueberry worldwide, which may impact the blueberry industry.

## 1. Introduction

The genus *Vaccinium*, in the Ericaceae family, is organized into sections comprised of important fruit crops including blueberries, which are mostly derived from the section Cyanococcus [1]. Blueberries are produced worldwide in at least 29 countries with the United States being the largest, accounting for almost 40% of the global blueberry production in 2019 [2]. Other major producers of blueberries in the world include Canada, Mexico, Peru, Poland and Spain. In the United States, the largest suppliers of fresh-market blueberries as of 2019 are the states of California, Georgia and Oregon [3].

The three species of blueberries in the section Cyanococcus produced commercially include highbush (*V. corymbosum* L.), lowbush (*V. angustifolium* Ait), and rabbiteye (*V. virgatum* Aiton) [4,5]. Highbush is the most popular commercially cultivated blueberry in the world, being produced in Argentina, Australia, Canada, Chile, New Zealand, United States, and several European countries [6,7]. The northern parts of the United States (Michigan, New Jersey, North Carolina, Oregon, and Washington) produce northern highbush blueberry (*V. corymbosum*). The southern United States (Florida, Georgia, and southern California) predominantly grow southern highbush blueberry (interspecific hybrids of *V. virgatum*, *V. corymbosum*, and *V. darrowii* Camp) [4,8].

*Vaccinium* spp. are exposed to existing and emerging viruses as a result of expanding acreage of blueberry plantation across the world, primarily in North America [9,10]. Since blueberry is cultivated in areas where there are wild *Vaccinium* spp., there is increasing risk of virus movement between wild and cultivated blueberries. This is theoretically possible because viruses can move from commercial cultivars to native species as well as from wild species to cultivated blueberries, causing the spread of existing and new viruses. The cost for cultivation and production of a perennial crop such as blueberry is significant, hence viral diseases in this crop can be economically devastating [10]. To date, blueberry is a known host to seventeen species of viruses (Table 1) [11]. Viral diseases usually produce a range of symptoms on plants, ranging from being completely asymptomatic to the extreme of causing plant death. The variation in the virus disease symptoms can be influenced by multiple factors such as the production systems, locations, and the type and age of the cultivars. However, it is also possible for viruses to cause no symptoms, which highlight the importance of knowing the current status of known and novel blueberry viruses as provided in this review. In addition, up-to-date data on the emerging blueberry viruses provided in this review can be incorporated into the blueberry certification programs to produce clean planting stocks, thus allowing a timely intervention of serious virus threats in blueberry production.

## 2. *Ilarvirus*

The genus *Ilarvirus* belongs to the family *Bromoviridae*, along with six other genera. Woody plants are the major type of plants that are infected by ilarviruses, which are spread mechanically by thrips feeding on virus-infected pollen grains or by transporting virus-infected pollen grains [50].

### Blueberry Shock Virus (BlShV)

Blueberry shock virus (BlShV) has been found in California, New York, Oregon, Pennsylvania and Washington, in the US, and Nova Scotia and British Columbia in Canada. During its first appearance in Washington in 1980, blueberry shock virus (BlShV) symptoms on blueberry were initially confused with blueberry scorch virus (BlScV) symptoms [12]. Although the plants appeared usual, BlShV-infected blueberries developed a second flush of foliage after blooming and fewer berries in late summer, while other symptoms of blighting on blossoms and leaves were similar to BlScV [51]. After 1–3 years, the flower and fruit of the infected blueberries seemed to be growing normally, with no additional symptoms [52].

BlShV virions are nonenveloped, quasispherical, and have a diameter of about 26–29 nm. They are made up of 180 CP subunits, each with a MW of about 27 kDa [12]. BlShV has a plus sense ssRNA genome of 8259 bp that is segmented into three sections. The virus is distantly related to prunus necrotic ringspot virus (PNRSV) and apple mosaic virus (ApMV), both members of subgroup 3 in the genus *Ilarvirus*, according to a serological test using indirect ELISA. BlShV is classified as an *ilarvirus* based on its physicochemical properties.

Like other ilarviruses, pollen is the main mode of transmission for BlShV, with a low level of transmission by seed [13]. The primary mechanism of BlShV transmission tends to be honeybees transferring BlShV-contaminated pollen from infected flowers to flowers on healthy plants. BlShV can be identified using ELISA or RT-PCR in buds early in the season and in leaf tissue as the season progresses until August in the Pacific Northwest [10]. If there is a combination of disease recovery and productivity recurrence, significant yield loss caused by BlShV-infected blueberry may be temporary due the reproduction of some infected berries [13,52]. When BlShV infection occurred in the field, it was managed by removing infected plants to minimize virus spread. However, since BlShV is pollenborne (virus is transmitted through pollens before symptoms develop) and is unequally distributed in blueberries during its early infection phase, this strategy will not be able to completely prevent the virus’s emergence. Since replantation to restoration of full yield may require 4–6 years following the removal of an infected field, the more cost-effective alternative is to let the virus run its course through a field, as suggested in the Pacific Northwest of the US [10]. For these reasons, the best practice would be to avoid introducing BlShV into new planting locations via nursery stock.

## 3. Unassigned Species of the Family *Closteroviridae*

The family *Closteroviridae* contain four genera while other species are yet to be assigned into a genus. Viruses in this family are commonly vectored by in a semipersistent manner by aphids, whiteflies, pseudococcid mealybugs or soft scale insects. Seed transmission is unknown, and experimental transmission via mechanical injection is difficult or impossible [53].

### Blueberry Virus A (BVA)

Blueberry virus A (BVA) was isolated for the first time from a highbush blueberry cultivar called ‘Spartan’ in Japan, and later was reported in Canada and the US [10,14]. Blueberry virus A was initially associated to leaf yellowing of blueberry, but graft transmission studies later revealed that BVA causes latent infection in blueberries. The complete BVA genome contains a 17-kbp ssRNA plus sense molecule, as well as 10 open reading frames. ORF 1a encodes a 338-kDa protein with motifs of papain-like proteases, MT, and HEL domains; ORF 1b contains RdRp domain; ORF 3 contains heat shock protein 70 homolog (HSP70h); ORF 4 encodes for a putative 60-kDa protein; ORF 5 encodes for a putative 23-kDa major CP; ORFs 6–9 encode proteins with unknown functions due to non-similarity to other virus proteins, which is a signature of closteroviruses [14]. Putative proteins of BVA (MT, HEL, RdRp, HSP70h, and CP) have the highest resemblance to other members of the *Closterovirus* genus, but they differ by more than 10%. Since BVA failed to form a clade with other closteroviruses based on phylogenetic analysis of the RdRp, HSP70h, and CP, it was proposed as a new species. Transmission of BVA has not been effective in a study using mechanical and aphid vector, thus its transmission mode is not yet known. Since BVA has not been associated with specific symptoms in single infections, the extent of economic damage to the blueberry industry is yet to be determined.

## 4. *Nepovirus*

The most common viruses that infect *Vaccinium* spp. belong to the genus *Nepovirus* from the *Secoviridae* family (Table 1). Nepovirus genomes are made up of two positive-sense, single-stranded RNA molecules, namely RNA1 and 2, that are encapsidated separately in isometric particles (M and B components), which are required for infectivity [54,55]. RNA1 is more conserved with size variation between 7.2–8.4 kb whereas RNA2 is more variable with size variation between 3.7–7.3 kb [56]. Nepoviruses are further divided into three subgroups (A, B and C) based on the length and arrangement of RNA2 as well as their sequence relatedness [57]. Both M and B components of subgroup A contain RNA2 of 3.7–4 kb, while only M component of subgroup B and C contains RNA2 of 4.4–4.7 kb and 6.4–7.3 kb, respectively [56,58]. The majority of nepoviruses infecting blueberry belong to subgroup C, except for tobacco ringspot virus, which belongs to subgroup A. Both the 5′- and 3′- UTRs are identical or almost identical between RNA1 and RNA2 in subgroup C nepoviruses but not identical in subgroup A. The conserved CG and GDD motifs serve as species demarcation criteria for members of the *Secoviridae* family, with 25% and 20% amino acid sequence divergence in the capsid protein (CP) and Pro-Pol regions, respectively [59]. The majority of nepoviruses infecting blueberry are known to be transmitted by nematodes, and by pollen and/or seeds.

### 4.1. Blueberry Latent Spherical Virus (BlSV)

Blueberry latent spherical virus (BlSV) was the first nepovirus isolated from asymptomatic highbush blueberry in Japan [15]. Graft inoculation of six blueberry cultivars with BlSV failed to produce any symptoms, although reverse transcription-polymerase chain reaction (RT-PCR) was able to detect this virus in all of them. Herbaceous hosts including *Chenopodium quinoa* Willd. quinoa, *Luffa cylindrical* (L) Roem, and *Nicotiana benthamiana* produced chlorotic spots in systemic leaves following mechanical inoculation of BlSV, suggesting that the virus is associated with latent infection of blueberry [15].

The virus belongs to subgroup C of nepoviruses based on the genome organization. It shares closest amino acid sequence similarities to the RdRp of peach rosette mosaic virus (57%) and CP of apricot latent ringspot virus (43%) [15]. The transmission mode and epidemiology of this virus have yet to be determined.

### 4.2. Blueberry Leaf Mottle Virus (BLMoV)

Symptoms of mottling and distortion on the leaves of highbush blueberries associated with blueberry leaf mottle virus (BLMoV) were first observed in 1977 in Michigan (USA) [16]. The virus also was found to occur in grapevine in New York (USA) but was reported by other group of researchers as a strain of GBLV at that time based on their distant serological relationship [16]. However, the characterization of GBLV genome has clearly shown that they are distinct species belonging to the same subgroup C of nepovirus [60]. The CP of BLMoV shared highest similarity with cherry leaf roll virus (CLRV) and ToRSV, indicating that it belongs to subgroup C of the nepovirus [61].

Although BLMoV is a member of nematode transmitted *Nepovirus* genus, it is spread randomly by honeybees through an infected pollen and possibly by seeds [17,18]. BLMoV-infected blueberry bushes show varying degrees of symptom severity depending on the cultivar, with symptoms in the cultivar ‘Rubel’ being the most extreme [62]. BLMoV was detected in commercial blueberry fields as well as wild *Vaccinium* spp. bushes surrounding the field, implying virus movement between cultivated and wild areas [63]. BLMoV can be detected in infected blueberry tissues using a commercial ELISA kit or RT-PCR but they are not fully accurate due to the lack of virus population data [10].

### 4.3. Cherry Leaf Roll Virus (CLRV)

The first occurrence of cherry leaf roll virus (CLRV) in blueberry was only reported recently in a southern highbush blueberry species (*Vaccinium darrowii* cv. Jubilee 83) in New Zealand in 2013, along with other isolates from various hosts [19,20]. CRLV was previously discovered in sweet cherry (*Prunus avium* L.) in England in 1955, and was later discovered in other European countries, North America, and other regions of the world, infecting a number of herbaceous and woody plants, including fruit trees of valuable horticultural crops [64,65,66]. CLRV causes chlorotic mottling and line-pattern symptoms, stunting and plant death symptoms in naturally infected *Rubus* sp. but the characteristic symptoms in blueberry were not known [67]. CLRV can potentially create a major impact on the agricultural sector due to its ability to infect a wide variety of hosts in different regions, threatening cash crop production in particular.

These virus isolates have long 3′ non-coding regions (1.5 kb) which are conserved between the 8 kb RNA1 and 7 kb RNA2 genomes, a feature seen in cherry and rhubarb isolates as well. Based on phylogenetic analysis of the CP and Pro-Pol regions, CLRV was confirmed to be closely related to other members of nepovirus-subgroup C [19,55]. Unlike other nematode-transmitted nepoviruses, the vector of this virus has yet to be determined, despite the fact that it can be transmitted mechanically and naturally via seed or pollen [55,68].

### 4.4. Peach Rosette Mosaic Virus (PRMV)

Peach rosette mosaic virus (PRMV) was first identified as a causal agent for rosette mosaic of peaches in the 1970′s, before being reported in grape and later in highbush blueberry [21,69,70]. The spread of PRMV is restricted to Michigan, New York and Ontario in North America [10]. Blueberry leaves infected with peach rosette mosaic virus (PRMV) bushes are distorted, malformed, and distributed unevenly across the plant. Only the blueberry cultivars ‘Jersey’ and ‘Berkeley’ were found to be infected by PRMV, where they were planted in a vineyard near a PRMV-infested site in Michigan (USA) [21]. Although the degree of yield losses caused by PRMV in blueberries is unknown, it is recognized as a threat to peach and grape crops [69].

A complete genome sequence of PRMV confirmed its status in subgroup C of *Nepovirus* genus [22,71]. PRMV is transmitted by two nematode species, *Xiphinema americanum* Cobband and *Longidorus diadecturus* Eveleigh and Allen, and is classified as a soilborne virus [23]. Serological and RT-PCR assays are commonly used to detect PRMV but indexing virus by *Chenopodium quinoa* provides a more effective detection than ELISA in some circumstances [72]. However, since these methods were developed using only one virus isolate, the detection results should be carefully interpreted.

### 4.5. Tobacco Ringspot Virus (TRSV)

A necrotic ringspot disease associated with tobacco ringspot virus (TRSV) in blueberry was first identified in New Jersey in the early 1960s [24,73]. The disease was then identified in six more states in the US (Arkansas, Connecticut, Illinois, Michigan, New York, Oregon and Washington) as well as two other countries (Canada and Chile) [25,62,74,75,76,77]. TRSV-infected blueberry plants produce symptoms including necrotic spots on leaves or flower buds, mosaic, leaf distortion, and shoot defoliation, leading to gradual decline in bush productivity in some cultivars or even plant death in others [62,76]. TRSV-affected cultivars include ‘Collins’, ‘Concord’, ‘Pemberton’, ‘Rubel’, and ‘Stanley’, although no cases have been documented in rabbiteye or lowbush blueberry [62].

TRSV is classified as a distinct member of Subgroup A nepovirus based on its serological relationship and the existence of RNA2 in both the M and B components of virus particles. TRSV is transmitted in blueberries by the nematode *X. americanum*, which is also seed transmitted in some other weeds and crops [78]. Plant sap containing TRSV can be mechanically inoculated into a variety of herbaceous hosts. The presence of TRSV in blueberry can be identified using RT-PCR or by ELISA, but can be difficult due to their unequal distribution in various sections of plant tissues [76].

### 4.6. Tomato Ringspot Virus (ToRSV)

Tomato ringspot virus (ToRSV) was first discovered in blueberries in 1972 (Caruso and Ramsdell, 1995), but it was only found in highbush varieties grown in the US (Washington, New York, Oregon, Pennsylvania), Canada, and Chile [25,62,74,75,76]. ToRSV-infected bushes showed similar necrotic ringspot symptoms caused by TRSV, with some variability in symptom severity between cultivars. Based on an observation in the 1980s in Oregon, the most susceptible highbush cultivars to ToRSV were ‘Berkeley’, ‘Earliblue’, ‘Pemberton’, and ‘Stanley’ [62].

ToRSV, like other blueberry-infecting nepoviruses, belongs to subgroup C of genus *Nepovirus* based on the almost inseparable middle and bottom components of its nucleoprotein, as well as the presence of high molecular weight RNA2 [78]. Complete sequences of ToRSV RNA1 and RNA2 further support the subgroup clustering [71,79]. ToRSV, like TRSV, is transmitted by *Xiphinema* spp. nematodes and is readily transmissible to a variety of herbaceous species by sap inoculation [25,78,80]. Since these two viruses are serologically unrelated, nucleic acid probes or ELISA may be used to distinguish them [10]. Caution should be taken in choosing appropriate detection test (RT-PCR or ELISA) due to the great differences of strains available in both viruses [10].

## 5. Unassigned Species in Family *Secoviridae*

The family *Secoviridae* contains five genera while other species are yet to be assigned into genus. The majority of known members are significant plant pathogens mostly infecting dicotyledonous plants. Most secoviruses are transmitted by known biological vector and can be spread easily via seed or pollen [56].

### Strawberry Latent Ringspot Virus (SLRSV)

Strawberry latent ringspot virus (SLRSV) had not been found in blueberry until recently in New Zealand in 2014, where it was discovered in *V. darrowii*, a new host of SLRSV [26]. SLRSV was previously discovered to naturally infect wild and cultivated rosaceous plants (e.g., black currant, cherry, plum, raspberry, and strawberry) in Scotland in 1964 [81], and has since been recorded in other continents around the world, including Asia, Oceania, and North America, in addition to European countries [82,83]. Symptoms of SLRV in blueberry have yet to be established, although it may cause varying degrees of decline and mottling, while being mostly asymptomatic in raspberry and strawberry plants [82].

Phylogenetic analysis of the CP region showed that the three SLRSV blueberry isolates from New Zealand, including one strawberry isolate from the United States, form a cluster with those from North America. This indicates that the virus was introduced to New Zealand by a single event. After being formerly assigned in the genus *Nepovirus* and *Sadwavirus*, SLRSV is currently placed in an unassigned genus of the family *Secoviridae* [71,84,85]. The transmission mode of SLRV in blueberry has yet to be determined, while mechanical inoculation of the virus into herbaceous hosts were not successful in previous study [26]. However, SLRSV is known to be transmitted by the nematodes *X. diversicaudatum* and *X. coxi*, and is seedborne in some plant species such as raspberry and celery [82].

## 6. *Sobemovirus*

The genus *Sobemovirus* belongs to the family *Solemoviridae*, along with two other genera. Each virus species has a limited natural host range, which includes monocotyledonous and dicotyledonous plant species. Sobemoviruses are spread primarily through mechanical injury of host plants and can be transmitted by a variety of insect species [86].

### Blueberry Shoestring Virus (BSSV)

Blueberry shoestring virus (BSSV), a sobemovirus, was first identified as the cause of shoestring disease in blueberry in New Jersey [28,31], and since then has spread across the states of Michigan, New Jersey, North Carolina, and Washington in the US, and Nova Scotia in Canada [51,87]. BSSV has a four-year latent cycle before causing symptoms on healthy blueberry plants in an infected area, and it spreads horizontally from bush to bush [62]. Current and one-year-old stems on infected blueberry plants have elongated (0.2 × 1.2 cm) reddish stripes that disappear as the growing season progresses. Flower ‘breaking’ can occur when longitudinal pink streaks appear on the petals. Infected leaves are narrow and curled, with the symptom of a shoestring. When the surface of immature berries on infected plants is exposed to light, it may turn prematurely reddish-purple [27]. Nine highbush cultivars were susceptible to the virus, whereas the cultivars ‘Blueray’ and ‘Atlantic’ showed field resistance to the disease [87]. BSSV is one of the most common viruses affecting cultivated highbush blueberries (*V. corymbosum*), with infected bushes losing up to 25% of their yield [88]. Shoestring disease caused a $3 million yield loss in a blueberry field in Michigan (USA) in 1981, making it one of the most economically significant diseases of highbush blueberries [87].

The BSSV virion is a nonenveloped isometric particle with a diameter of 28 nm. It has a single-stranded RNA genome with four putative ORFs (ORF1, ORF2a, ORF2b, and ORF3). The 15.4 kDa movement protein, a 65.55 kDa polyprotein protein (Protease-VPg), a 62.22 kDa RNA-dependent RNA polymerase protein, and a 27.85 kDa coat protein were encoded by ORF1 (nt No. 85-492), ORF2a (nt No. 462-2204), ORF2b (nt No. 1796-3400), and ORF3 (nt No. 3186-4001), respectively [89]. BSSV is classified as a sobemovirus based on its physicochemical properties and genomic sequence structure [27,89,90]. Aphids (*Illinoia pepperi*) vectored the transmission of BSSV into blueberry plants in a persistent, circulative manner [87]. Although BSSV cannot be inoculated mechanically into herbaceous plants, the virus can be inoculated mechanically into blueberry seedlings or rooted softwood cuttings [27]. Commercial ELISA kits or RT-PCR can be used for the detection of BSSV in infected blueberry plants. Furthermore, management control of BSSV through rogueing alone is ineffective due to the long latent period of BSSV in infected blueberries prior to symptom appearance. Therefore, the use of virus-free planting material and timely insecticide applications, in addition to removing infected plants, will help control the spread of shoestring disease.

## 7. *Blunervirus*

The genus *Blunervirus* has been recently classified in the family *Kitaviridae*.

### Blueberry Necrotic Ring Blotch Virus (BNRBV)

**Figure 1:**
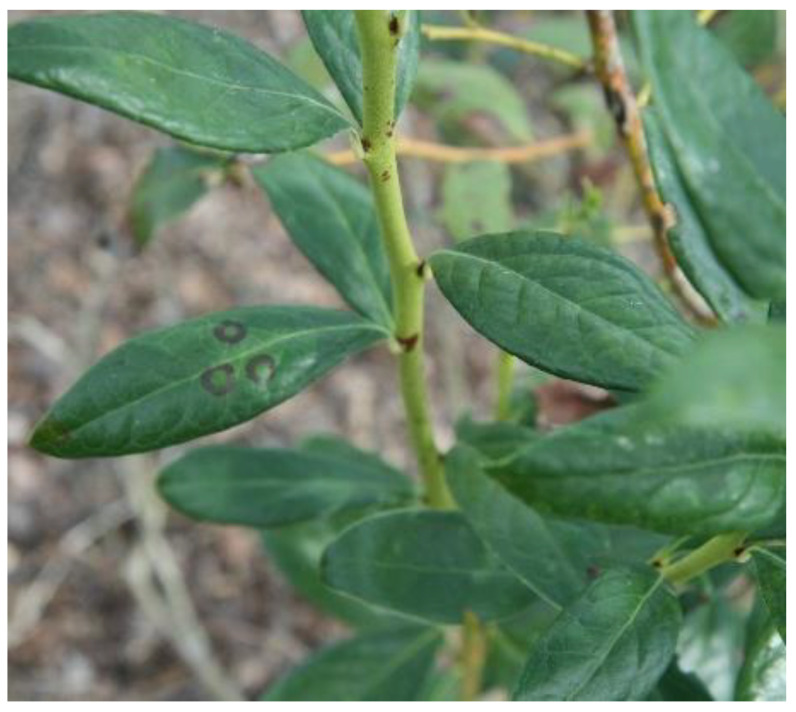
Cultivated blueberry plant in a farm in Florida showing Blueberry necrotic ring blotch virus (BNRBV) symptomatic leaves with necrotic rings with green cores.

Blueberry necrotic ring blotch virus (BNRBV) was first discovered in southern highbush blueberries showing blueberry necrotic ring blotch symptoms in Georgia (USA) in 2006 [10]. Since then, the disease has been documented in blueberry fields in the states of Florida, Mississippi, North Carolina, and South Carolina in the US. Northern highbush blueberries and native rabbiteye blueberries (*V. virgatum*) have not been infected with BNRBV, while southern highbush cultivars are known to be susceptible. Infected blueberries had distinct necrotic rings with green cores, but when the rings fused, they resembled the symptoms of fungal diseases (Figure 1). Early defoliation can occur in severely infected bushes, which can be mistaken for Septoria leaf spot disease. Unlike blueberry red ringspot virus (BRRV), which usually only affects the upper leaf surface and stems, BNRBV infected leaves may have necrotic rings on both the upper and lower surfaces, but the stems do not show symptoms.

The BNRBV genome is approximately 14 Kb long and consists of four RNA segments with seven ORFs (RNA1, 2, and 4 have one ORF each, while RNA3 has up to five ORFs) [29,30]. RNA1 expresses methyltransferase (MTR), cysteine-protease (C-Pro), and helicase (HEL) from a putative 215 kDa protein, RNA2 expresses HEL and RdRp from a putative 130 kDa protein, RNA3 expresses up to five small proteins with unknown functions, and RNA4 expresses a 34 kDa protein with conserved motifs from the 3A movement protein superfamily [29]. The amino acid relatedness of different BNRBV genome segments to the alphavirus-like supergroup protein domains that are conserved among RNA viruses was discovered via protein analysis. Based on phylogenetic analysis of the RdRp, BNRBV belongs to the same clade as virus species from the genera *Cilevirus* and *Higrevirus*, but it was recently assigned to a new genus, *Blunervirus* [29,91].

Based on its protein sequence similarity to citrus leprosis virus, BNRBV is most likely transmitted by an eriophyid mite. Transmission studies of BNRBV using eriophyid mite are currently being performed in Florida and Georgia to test this hypothesis [92,93]. BNRBV has recently been shown not to spread through vegetative propagation, leading to the conclusion that BNRBV-infected southern highbush plants do not develop systemic infection [93,94]. BNRBV can be detected in symptomatic tissue using molecular techniques like RT-PCR.

## 8. *Ophiovirus*

*Ophiovirus* is currently the only genus recognized under the family *Aspiviridae* (formerly *Ophioviridae*). Trees, shrubs, vegetables, and bulbous or corm-forming ornamentals, including monocots and dicots, are natural hosts for aspiviruses. Four of the seven species in the family are known to be soil-transmitted [95].

### Blueberry Mosaic Associated Virus (BlMaV)

Although mosaic disease of blueberry was initially thought to be a physiological disorder, it was discovered to be related to viruses in the 1950s due to its graft-transmissibility [31]. After its initial report, the disease has been discovered in various parts of North and South America, as well as Asia, Europe, New Zealand, and South Africa [9,10,96]. Blueberry mosaic has been found in blueberry cultivated areas in the US, including Indiana, Michigan, New Jersey, New York, Oregon, Washington, Kentucky and most recently, Florida [97,98,99]. The highbush cultivars ‘Bluecrop’, ‘Cabot’, ‘Concord’, ‘Earliblue’, ‘Jersey’, ‘Pioneer’, ‘Rubel’, and ‘Stanley’ are the most susceptible to blueberry mosaic disease. Mosaic diseases cause blueberry bushes to have bright yellow to yellow-green colorations on their leaves, resulting in mosaic and mottling patterns that sometimes turn pink. Symptoms can occur in patches or may be widely spread across the infected bush, and they can appear at any time of year [97]. Despite the lack of data on the economic impact of blueberry mosaic disease, infected bushes have been reported to have lower yields and poor-quality berries with delayed maturity [62].

The causative agent associated with blueberry mosaic disease has yet to be confirmed due to the difficulties in characterizing the agent. However, blueberry mosaic associated virus (BlMaV) has been discovered in mosaic-affected blueberries as well as asymptomatic plants in North America. It has been proposed as a possible cause of blueberry mosaic disease [32]. BlMaV has been thought to only infect the highbush and a lowbush dryland blueberry (*V. pallidum*), until it was recently reported in wild highbush, *V. corymbosum* in Florida [62,97,99].

BlMaV is proposed as a new member of the only genus in the family *Ophioviridae,* the *Ophiovirus*, based on phylogenetic analysis of the RdRp region. Based on phylogeny clustering and genome arrangement, BlMaV is closely related to citrus psorosis virus (CPsV) [100]. The naked and flexuous nucleocapsids of ophiovirus virions have a diameter of about 3 nm and form kinked circles of at least two different contour lengths, the shortest being about 760 nm [101]. The genome of BlMaV is made up of three negative-strand ssRNA segments (RNAs 1–3) that encode for four proteins on the viral complementary strand. Two ORFs in RNA1 encode for a 272 kDa RdRp and a 23 kDa protein of unknown function. The ORFs on RNAs 2 and 3 encode a 58-kDa movement protein (MP) and a 40-kDa nucleocapsid protein (NP), respectively.

Similar to its closest relative, CPsV, the natural vector of BlMaV is still unknown although other ophioviruses are transmitted via fungal spores, suggesting that the BlMaV could have a similar soilborne vector [102]. The practice of vegetative propagation in blueberry cultivation can provide a means for virus spread through propagation of infected stock in producing nursery plants. Hence, a fast and reliable detection assay needs to be developed for BlMaV screening in nursery and field.

## 9. *Carlavirus*

*Carlavirus* belongs to the family *Betaflexiviridae*. Some carlaviruses can infect a broad variety of experimental hosts, although some have limited natural host ranges. The majority of species are nonpersistently transmitted by aphids and are mechanically transmissible.

### Blueberry Scorch Virus (BlScV)

**Figure 2:**
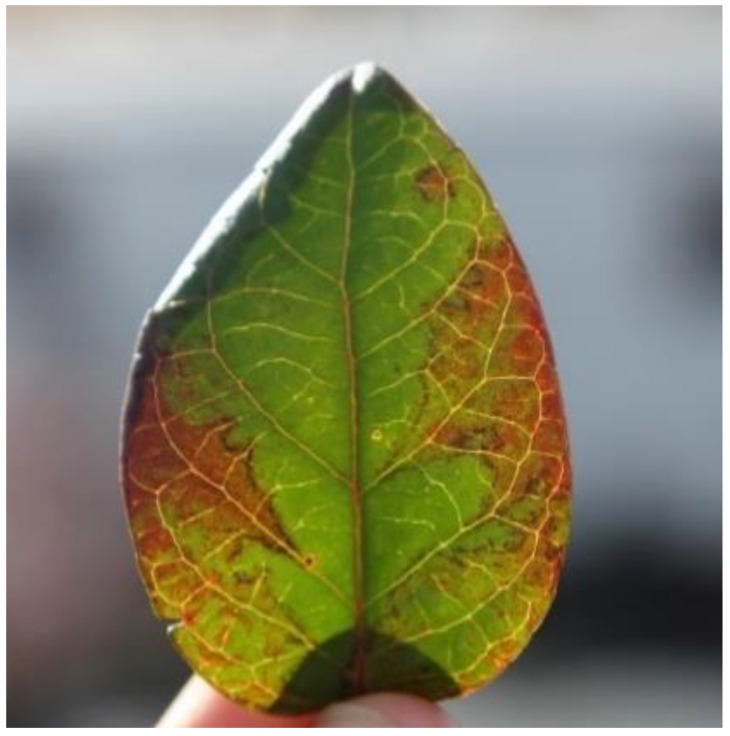
Blueberry leaf showing red line and ring patterns typical of virus symptoms (Photo courtesy Philip Harmon, University of Florida).

Blueberry scorch and Sheep Pen Hill diseases were discovered on highbush blueberries in the states of Washington and New Jersey in the USA in the 1980s. Different strains of the same BlScV virus, a carlavirus, were identified as the causal agent [35,103,104,105,106]. BlScV has since been documented in Canada, Germany, Italy, The Netherlands, Poland, United States (Connecticut, Massachusetts, Michigan, New Jersey, Oregon, and Washington) and recently in China [37,38,39,40,107,108]. The expression of BlScV symptoms is influenced by a number of factors, including the season of occurrence, cultivar, and virus strain. BlScV symptoms include full blighting of blossoms, necrosis of young foliage, and stem dieback in susceptible cultivars, though some cultivars may appear asymptomatic [36]. Furthermore, some infected cultivars can produce a red line shape or chlorosis at the leaf margins (Figure 2) [51]. Scorched flowers can either stay on the bushes until the next season or fall off right away [10]. Symptoms appear after many years upon infection, suggesting a latent phase in the disease’s growth until it spreads across the entire plant [51]. In certain cultivars, such as ‘Berkeley’, the disease can reduce yields and ultimately destroy the plant, while others can continue to produce for a while [10,36]. Blueberry scorch disease has been observed in commercial plantings of northern highbush blueberries, with more than 15 cultivars susceptible to BlScV [36].

BlScV virions are nonenveloped, flexuous particles (690 nm long × 14 nm wide) made up of a 33,500 kDa capsid protein encasing an 8.5 kb positive-sense ssRNA [35,106] (Martin and Bristow, 1988; Cavileer et al., 1994).The BlScV genome contains six ORFs. ORF 1 expresses a putative polymerase of 223 kDa with motifs for methyltransferase, NTP-binding/helicase, and RdRp; ORFs 2–4 encode for the triple gene block proteins (25, 12, and 7 kDa) involved in viral movement, followed by ORFs 5 and 6 that encode for the CP and cysteine-rich protein, respectively [106]. The virus was placed in the family *Alphaflexiviridae*, along with other members of the genus *Carlavirus*, based on the organization and sequence of the viral genome as well as serological relationships [84]. Sequence comparisons of BlScV strains from Washington and New Jersey indicated that they are distinct strains of more than 10% divergence, although sequence analysis of BlScV strains at the 3′-terminal and CP regions revealed that they are more closely related to potato virus S and lily symptomless virus [106].

Although the significance of aphid *Ericaphous fimbriata* in natural disease spread is uncertain, it has been shown to inefficiently transmit BlScV in a nonpersistent manner [9,36]. BlScV can also be graft transmitted to many half-high and southern blueberry cultivars or transmitted mechanically using infectious transcripts [36,109]. Due to the unreliability of the host indicator protocol and the absence of symptoms in some plants, diagnostic techniques based on serology or nucleic acid are needed for BlScV detection [108]. Double antibody sandwich ELISA (DAS-ELISA), the most effective and inexpensive technique has been used for mass-detection of BlScV in blueberries [110,111]. Nonetheless, since the source of tissue and sampling date influenced the results of DAS-ELISA, the RT-PCR approach provides higher sensitivity for BlScV detection [110]. Since blueberry scorch disease has been reported to cause significant yield loss, it is critical to monitor virus spread by including symptomless mother plants for virus testing, as BlScV can be spread via infected nursery stock [112].

## 10. *Vitivirus*

*Vitivirus* is another genus in the family *Betaflexiviridae* containing virus species infecting blueberry. Pseudococcid mealybugs, soft scale insects, and aphids are the natural carriers of vitiviruses.

### Blueberry Green Mosaic-Associated Virus (BGMaV)

The presence of an novel vitivirus was recently discovered by high throughput sequencing of a blueberry plant (cultivar ‘Bluecrop’) with green mosaic symptoms collected from Burlington County, New Jersey [41]. The new vitivirus, tentatively named as “blueberry green mosaic-associated virus” (BGMaV), was the only virus discovered in the symptomatic plant, and it was later observed in many other plants with green mosaic symptoms [113].

BGMaV has a vitivirus-like genome structure, containing a ~7.5-kbp ssRNA molecule plus sense with five ORFs and a polyadenylated 3′ terminus. BGMaV’s ORF1 encodes a putative viral replicase (196K); ORF2 expresses a 16-kDa protein with no known function. Movement protein, CP, and nucleic acid binding protein are encoded by ORFs 3–5, respectively. BGMaV was inferred as a novel vitivirus based on phylogenetic analyses of the CP and conserved motifs of RdRp (Thekke-Veetil and Ho, 2019). The discovery of BGMaV necessitates its inclusion in blueberry certification programs in order to ensure consistent propagation and clean source of plant stocks.

## 11. *Tepovirus*

The tepoviruses are members in the family *Betaflexiviridae*, containing only two approved species (*Potato virus T* and *Prunus virus T*). The viruses in this genus has no recognized vector yet.

### Blueberry Virus T (BlVT)

A novel tepovirus, tentatively named as blueberry virus T (BlVT), has been recently discovered through metagenomics approach in blueberry cultivar ‘Gulf Coast’ and ‘Windsor’ from Island Grove, Florida [99]. The specific virus symptoms caused by BlVT, however, could not be established yet in blueberry since it was detected in only 3 of the 20 samples tested.

BlVT genome is similar to those of tepoviruses, consisting of three overlapping ORFs encoding for RdRp (5457 nt), MP (1146 nt), and CP (663 nt), which makes up the 7.2-kbp genome. Phylogenetic analysis of the putative RdRp and CP encoded by BlVT suggested that this virus belongs to the genus *Tepovirus*. Similar to BGMaV, the discovery of BlVT has prompted the need to incorporate this virus in the blueberry certification program for the production of BlVT-free blueberry plants.

## 12. *Amalgavirus*

The genus *Amalgavirus* is the only genus containing plant viruses belonging to the family *Amalgaviridae*, a recently described group of dsRNA viruses. There were only four approved species in this genus, including blueberry latent virus (BlLV).

### Blueberry Latent Virus (BlLV)

When a new disease known as blueberry fruit drop disease was detected in the Pacific Northwest (Oregon, Washington, and British Columbia) in the US in the early 2000s, blueberry latent virus (BlLV) was unwittingly discovered [9,42]. BlLV has a broad geographic distribution, as it was initially found in nonsymptomatic and symptomatic plants from Arkansas, Florida, Michigan, New Jersey, and the Pacific Northwest, as well as blueberry germplasm from North America [10,42,99]. It was later shown, however, blueberry infected with single infection of BlLV does not cause any striking symptoms [42]. Regardless of the absence of symptoms, its role in mixed infection with other viruses is yet to be determined.

Despite the fact that blueberry fruit drop was later discovered to be unrelated to BlLV, the virus was studied further, resulted in the isolation of a 3.5 kb dsRNA molecule belonging to a virus now known as BlLV [42]. BlLV’s genome structure is similar to that of southern tomato virus (STV), which has two partly overlapping ORFs encoding a replicase and an unknown protein [42,114]. Although the genome organization of BlLV is similar to those of totiviruses, its RdRp has been linked to members of the *Partitiviridae* family [42,115]. As a result, BlLV is now classified in a new genus *Amalgavirus* in the *Amalgaviridae* family, with STV as the type species [116]. It was also discovered that the virus has a rather stable population structure when partial and full sequences of BlLV isolates from Japan and the United States were compared, with less than 0.5 percent diversity between isolates from the two countries [10,42,117].

BlLV is transmitted efficiently by seeds although in the absence of movement protein, implying that it replicates in its host during cell division. The lack of symptoms in certain BlLV-infected highbush cultivars has led to the conclusion that the presence of these viruses in blueberries is not a cause for concern yet.

## 13. *Soymovirus*

The genus *Soymovirus* belongs to the family *Caulimoviridae*, with four approved species reported so far. The host range of soymoviruses are limited to one or two plant families and their vectors are still not known.

### Blueberry Red Ringspot Virus (BRRV)

**Figure 3:**
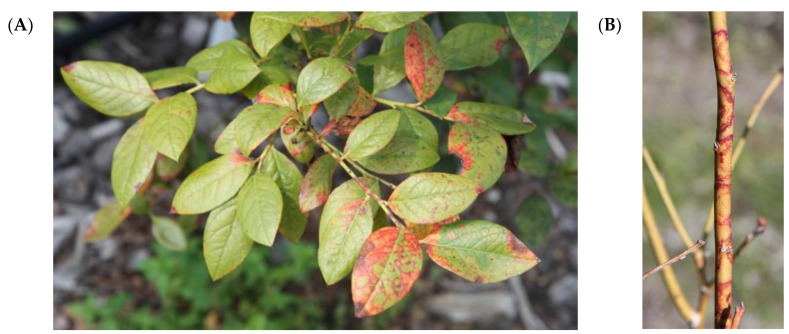
Symptoms of BRRV on leaves and stem of blueberry plants. (**A**) Red blotches on blueberry leaves. (**B**) Red ring spots on stem. (Photo courtesy Philip Harmon, University of Florida).

Red ringspot disease originally was described in New Jersey with associated symptoms observed on highbush blueberry in the 1950s [43] and since then has rapidly expanded to other states in the US, as well as other countries including Czech Republic, Japan, Korea, Poland, Serbia and Slovenia [10,34,43,44,45,46,47,48,49]. Symptoms are usually seen in late summer and early fall on older leaves as red blotches resulted from the coalescence of round red spots (Figure 3A). Also common is the appearance of pale green lesions surrounded by red rings with a diameter of 2–3 mm and 5–15 mm on leaves and stems (Figure 3B), respectively [118]. The red spots on leaves are a typical disease diagnostic characteristic that is commonly observed on the upper leaf surface, but both sides of the leaves can be symptomatic depending on cultivar. Sometimes the red rings can also be visible on ripening fruit but disappear as the fruit ripens. Infected fruits can also become distorted and unmarketable, such as in the case of cultivar ‘Ozarkblue’ [10].

BRRV, the causal agent for red ringspot disease in blueberry, is a paratretrovirus that belongs to the genus *Soymovirus* in the family *Caulimoviridae* [44,119]. BRRV has an 8.3 kb circular double-stranded DNA genome encapsidated in a nonenveloped, icosahedral particle with a diameter of 42–46 nm that can exist as a virion or form inclusion bodies in the nucleus or cytoplasm, respectively [45,120]. Members of the genus *Soymovirus* have a genome that encodes for eight proteins with discontinuities in both the transcribed and nontranscribed strand. These gaps are sealed upon infection of the virus into the host cell. The closed dsDNA is then transcribed into mRNA in the nucleus by host DNA-dependent RNA polymerase. This serves as a template for synthesis of viral proteins and reverse transcribed into new copies of dsDNA genomes. New virions are released following encapsidation of the new dsDNA genomes [121].

Reliable diagnostic tests are available for BRRV and commonly involve conventional polymerase chain reaction (PCR). BRRV specific primers from New Jersey are used since no reliable detection is available for a routine enzyme linked immunosorbent assay (ELISA) test [122], and the virus is not readily sap-transmissible [62]. Another test used to validate BRRV is visualization of virus particles or inclusion bodies by doing transmission electron microscope (TEM) on the infected plant. Virus particles will appear as icosahedral symmetry shape with 42–46 nm diameter without envelope. However, PCR is routinely used since TEM is more time consuming and requires special equipment and skill.

To date the vector for BRRV and other members of the *Soymovirus* genus remain unknown, though the red ringspot disease can be transmitted through grafting and softwood cuttings [43,94]. Infected plants used in propagation can be the source of virus spread due to symptoms variation within cultivars from softwood cuttings and undetectable symptoms on hardwood cuttings [10]. Although aphids and mealybugs are proposed to be responsible for BRRV transmission, there is a lack of experimental or other existing evidence that can support this assumption [122]. The unsuccessful identification of BRRV vector suggests the likelihood of vegetative propagation as the mode of virus spread. This situation, however, has restricted the epidemiology of red ringspot disease, thus confounding the control of BRRV. A recent finding showed that BRRV in the southeastern United States does not cause significant yield loss due to the relatively benign infection in southern highbush blueberry cultivars ‘Star’ and ‘Jewel’, while surprisingly may cause early ripening of berries in ‘Star’ [123].

## 14. Conclusions

North America used to be the center for blueberry production, accounting for over 80% of global production from 2008–2010 [124]. The production of blueberry has now been expanded in other parts of the world, to the point that North American production accounts for less than half of global production [125]. The rapid growth in blueberry production around the world has led to the increase in foreign trade due to the exchange of plant stock or germplasm between blueberry nurseries in different regions. The trade of planting materials across blueberry production regions possibly increases the risk of viral movement in these plants. Moreover, commonly used practices in blueberry production, such as cuttings, grafting and interspecific hybridization, may provide means for viruses to spread. Another pathway that potentially increases viral movements in blueberry plants is the lack of virus screening prior to the use of native wild blueberries in the development of new cultivars. The establishment of new blueberry cultivation areas near wild plants of the same and related species, for instance in Florida, could further contributes to the spread of viruses in these plants.

A comprehensive review of blueberry and cranberry viruses was published almost a decade ago [10]. Although viruses are currently not a major threat to the global blueberry production, the number of viruses infecting blueberries are increasing with the expansion of growing regions. The advent of high-throughput sequencing and bioinformatics have undoubtedly facilitated the discovery of viruses in blueberry, as shown by characterization of the first complete BRRV genome from Florida [126], as well as the identification of two novel virus species in the family *Betaflexiviridae*, in within the last 3 years. Interestingly, the metagenomics study which has uncovered the new tepovirus have also led to the discovery of BlLV and BlMaV for the first time in Florida, and the first to demonstrate the occurrence of BlMaV in wild highbush [99].

Blueberry producers should be aware of the viruses present in their respective area and develop a proper control strategy to prevent an outbreak. In North America, a total of nine viruses (BlMoV, BlScV, BlShV, BNRBV, BRRV, BSSV, BVA, ToRSV, and TRSV) have been reported as high risk due to their frequent occurrence and widespread in different production regions [127]. In contrary, only two viruses have been reported in other countries such as Japan and New Zealand, and recently one from China, implying that blueberry viruses may not be a major concern in these regions. Overall, this comprehensive review presented the current status of known and new emerging blueberry viruses worldwide, which is crucial for global blueberry producers to prevent tomorrow’s virus problem that may be lurking in fence rows and natural areas today.

## Figures and Tables

**Table 1:** Virus species reported in *Vaccinium* spp. in United States and around the world.

Type of Genome	Family	Genus	Virus Species	Geographic Regions	Transmission Mode	References
ssRNA (+) ^1^	*Bromoviridae*	*Ilarvirus*	*Blueberry shock virus*	Canada and USA	Pollen and seed	[12,13]
	*Closteroviridae*	Unassigned	*Blueberry virus A*	Canada, Japan and USA	Unknown	[14]
	*Secoviridae*	*Nepovirus*	*Blueberry latent spherical virus*	Japan	Unknown	[15]
			*Blueberry leaf mottle virus*	USA	Pollen	[16,17,18]
			*Cherry leaf roll virus*	New Zealand	Pollen and seed	[19,20]
			*Peach rosette mosaic virus*	Canada and USA	Nematode	[21,22,23]
			*Tobacco ringspot virus*	Canada, Chile and USA	Nematode	[24,25]
			*Tomato ringspot virus*	Canada, Chile and USA	Nematode	[25]
		Unassigned	*Strawberry latent ringspot virus*	New Zealand	Unknown	[26]
	*Solemoviridae*	*Sobemovirus*	*Blueberry shoestring virus*	Canada and United States	Aphid	[27,28]
	*Kitaviridae*	*Blunervirus*	*Blueberry necrotic ring blotch virus*	United States	Unknown	[29,30]
ssRNA (−) ^2^	*Aspiviridae*	*Ophiovirus*	*Blueberry mosaic associated virus*	Argentina, Canada, Chile, Europe, Poland, Serbia, South Africa and USA.	Unknown	[31,32,33,34]
	*Betaflexiviridae*	*Carlavirus*	*Blueberry scorch virus*	Canada, China, Germany, Italy, The Netherlands, Poland and United States	Aphid	[35,36,37,38,39,40]
		*Vitivirus*	Blueberry green mosaic-associated virus	USA	Unknown	[41]
		*Tepovirus*	Blueberry virus T	USA	Unknown	[11]
dsRNA ^3^	*Amalgaviridae*	*Amalgavirus*	*Blueberry latent virus*	Canada, Japan, USA	Seed	[42]
dsDNA (RT) ^4^	*Caulimoviridae*	*Soymovirus*	*Blueberry red ringspot virus*	Czech Republic, Japan, Korea, Poland, Serbia, Slovenia and USA	Unknown	[34,43,44,45,46,47,48,49]

^1^ ssRNA (+): positive-sense single-stranded RNA; ^2^ ssRNA (−): negative-sense single-stranded RNA; ^3^ dsRNA: double-stranded RNA; ^4^ dsDNA(RT): double-stranded DNA reverse-transcribing.

## Data Availability

Not applicable.
